# Pharmacovigilance workflow in Europe and Italy and pharmacovigilance terminology

**DOI:** 10.1007/s11096-018-0711-z

**Published:** 2018-08-09

**Authors:** Paolo Baldo, Sara Francescon, Giulia Fornasier

**Affiliations:** Pharmacy Unit, CRO Aviano IRCCS, National Cancer Institute, Aviano, Italy

**Keywords:** Adverse drug reaction, Definitions, Pharmacovigilance system, Signal, Alerts, Terminology

## Abstract

The terminology used in pharmacovigilance can cause confusion, because there are similar terms that describe different phenomena (e.g. adverse reactions, adverse drug reactions, and side effects). Incorrect use of terminology can have negative effects on the reporting of adverse drug reactions and on the interpretation of these reports. To explain the most common terms used in pharmacovigilance, this article first describes the pharmacovigilance workflow process in the European Union and, as an example, in Italy. Then, the article reviews common pharmacovigilance terms.

## Introduction

As Socrates is thought to have said, “The beginning of wisdom is the definition of terms”.

Definitions are essential for understanding the meaning of words and how to use them correctly, and this is ever more true for scientific terminology. In pharmacovigilance, there are many similar-sounding terms with unique yet complex definitions (e.g. adverse reaction, adverse drug reaction, side effect, serious adverse event, and unexpected adverse event). To complicate matters further, this field involves the efforts of, and interactions among, operators from different professions: doctors, pharmacists, nurses, and more [1, 2]. Misuse of this terminology can lead to misunderstanding, with potentially serious consequences on pharmacovigilance activities. Accurate use of this terminology is important so that regulatory agencies can take measures to ensure the safety of medicines [3]. Moreover, a basic knowledge of pharmacovigilance systems and terms will be useful for healthcare professionals and may help reduce the phenomenon of under-reporting of adverse drug reactions (ADRs) [2].

Under-reporting of ADRs by health care professionals can be the consequence of poor knowledge of pharmacovigilance terms and processes [2, 4]. If all health care operators know which ADRs to report and how to report them, the problem of under-reporting may improve. A first step in this direction is understanding pharmacovigilance terminology and processes. Therefore, in this article of this special issue on pharmacovigilance, we describe the pharmacovigilance processes in Italy and the European Union (EU), and define the most common terms used to describe ADRs. We take as example the Italian context to highlight the close relationships between the EU and national systems.

## Pharmacovigilance workflow: Italy and the EU

**Fig. 1:**
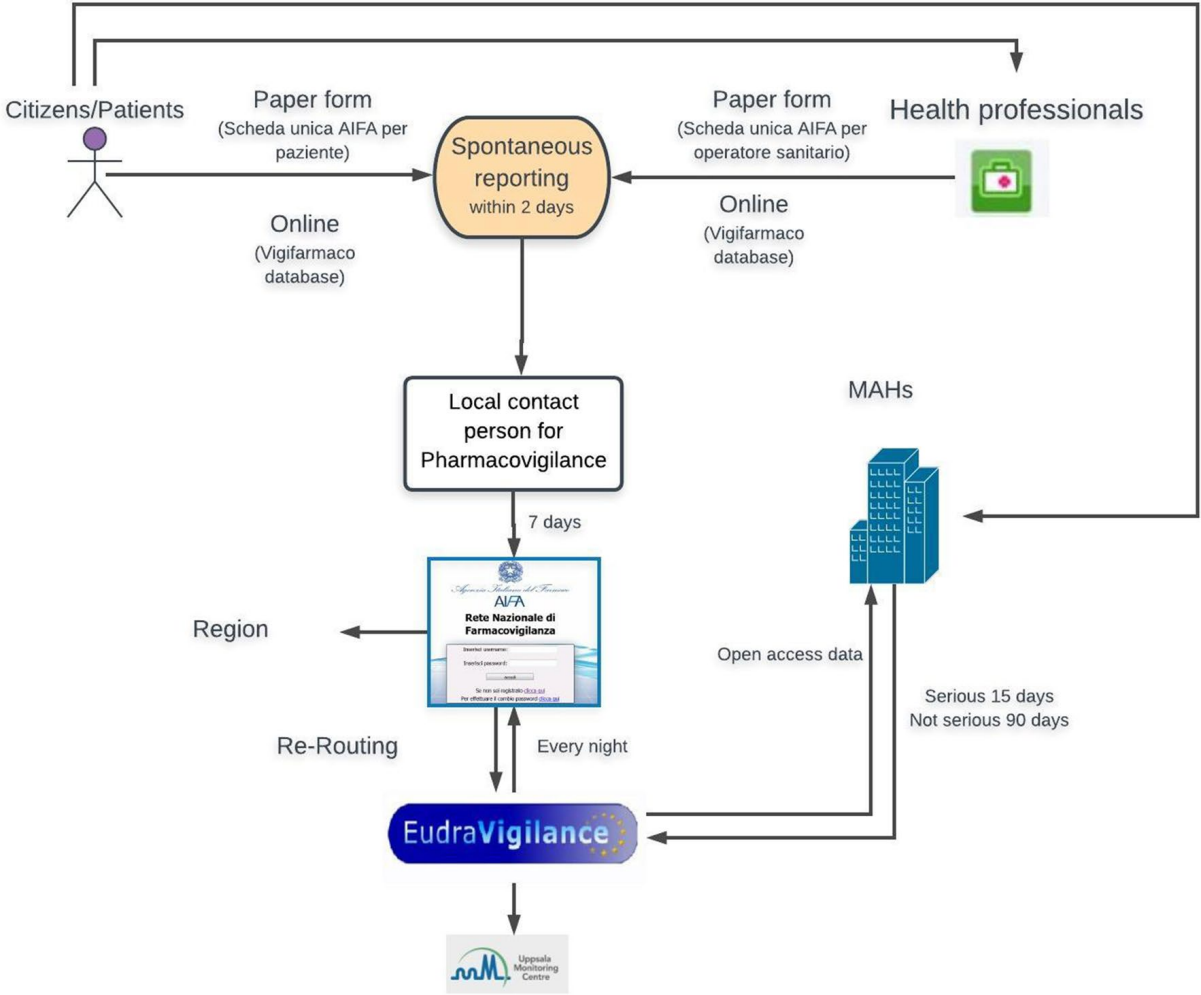
Steps of the Italian pharmacovigilance process. *MAHs* marketing authorization holders (adapted and modified from AIFA [7])

When a drug is suspected of having caused an adverse reaction, the pharmacovigilance system is activated by a *spontaneous report* (Fig. 1). As defined by the European Medicines Agency (EMA), a spontaneous report is a voluntary, unsolicited notification by a patient, a health professional or anyone else to medicine regulators to alert them about a possible adverse drug reaction [5]. Whether the observed reaction is due to the drug or not will then be determined by regulatory authorities in a procedure called causality assessment.

Spontaneous reports are sent to regulatory agencies. In Italy (Fig. 1) [6, 7], they are first sent to the local contact person for pharmacovigilance, who is listed on the website of the Italian Medicines Agency (Agenzia Italiana del Farmaco, AIFA) and may work in a local hospital or district health agency. This person has 7 days to insert a spontaneous report into AIFA’s pharmacovigilance network (Rete Nazionale di Farmacovigilanza, RNF) and also send it to the regional pharmacovigilance system. RNF is linked to EudraVigilance, the EMA’s pharmacovigilance network. EudraVigilance has a database of information on suspected adverse reactions to medicines that have been authorized for sale or are being studied in European clinical trials [8]. EudraVigilance sends spontaneous reports to the pertinent pharmaceutical company that is the marketing authorization holder (MAH), so that it can investigate the problem [5]. MAHs also send any reports they receive from patients to EudraVigilance. In this case, EudraVigilance sends the reports to AIFA (called “re-routing”). Finally, spontaneous reports are sent by EudraVigilance to the World Health Organization-Uppsala Monitoring Centre.

Spontaneous reports differ from *solicited reports*, which come from organized data collection systems (e.g. clinical trials, non-interventional studies, registries, and health institutions). In these cases, whenever an ADR is suspected, a causality assessment is enacted; only when a high causal association between the adverse reaction and the drug is established is a solicited report prepared [9, 10].

Reports of ADRs (both spontaneous and solicited) are essential, because they indicate the need for further investigations to confirm the association with the drug, and may trigger alarm signals. *Signals* are defined by EMA as “Information that arises from one or multiple sources (incl. observations and experiments), which suggest a new potentially causal association, or a new aspect of a known association, between an intervention and an event or set of related events, either adverse or beneficial, that is judged to be of sufficient likelihood to justify verificatory action” [11].

The analysis of signals is extremely important. Detection of a signal does not necessarily mean an ADR occurred. Signal detection is the first step in determining if a new, previously unknown ADR occurred, or if there is a change in frequency of an ADR already described in the Summary of Product Characteristics (SmPC) document. The SmPC is a legal document approved during the marketing authorization process; meant for healthcare professionals, it describes how to use a drug based on information from clinical trials [12].

**Fig. 2:**
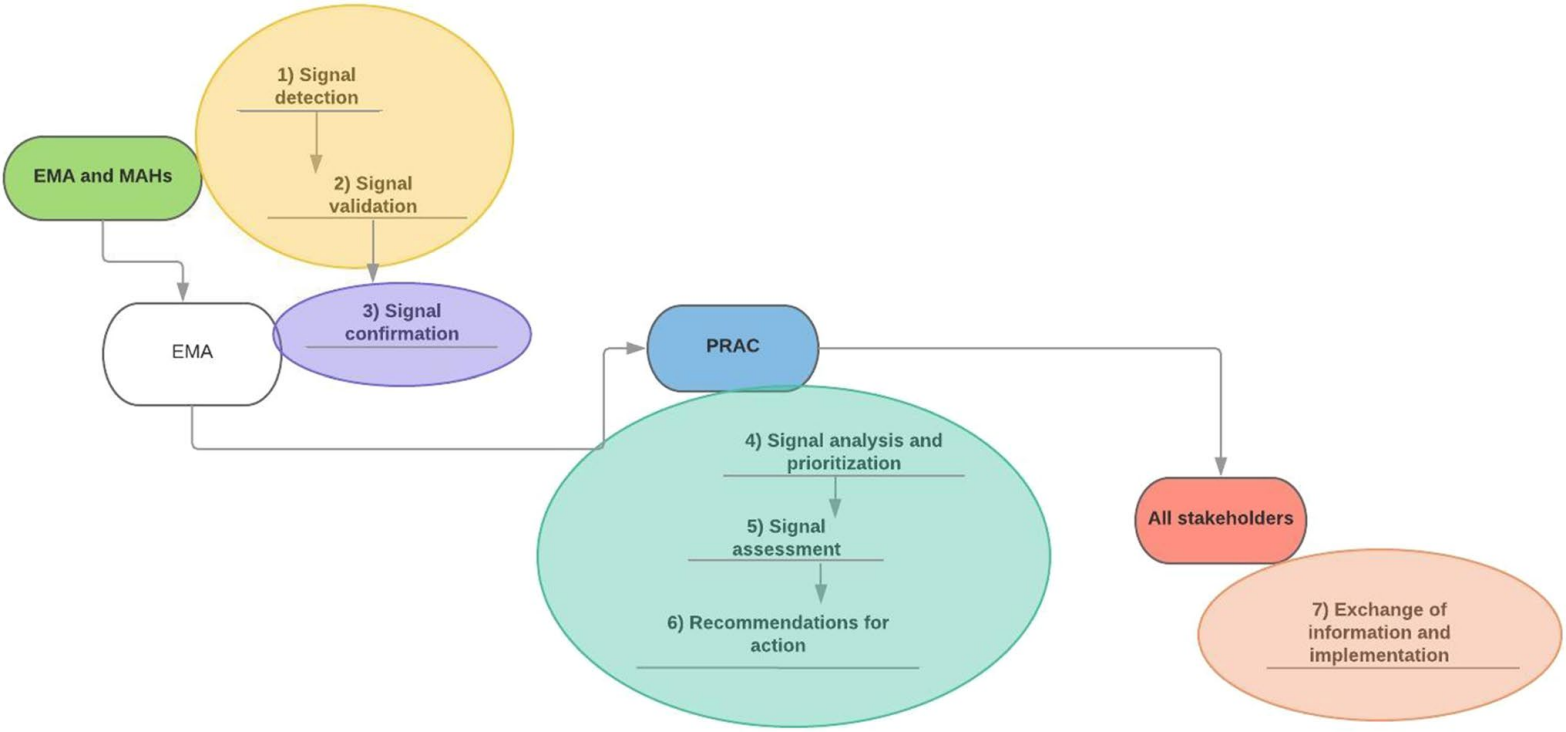
Steps of the EU signal management process in pharmacovigilance. *EMA* European Medicines Agency, *MAHs* marketing authorization holders; *PRAC* Pharmacovigilance Risk Assessment Committee (adapted and modified from AIFA [7])

As explained in Module IX of EMA’s Good Pharmacovigilance Practices [13], the steps of the signal management process are detection, validation, and confirmation by EMA and the MAHs (Fig. 2). Then the EMA’s Pharmacovigilance Risk Assessment Committee (PRAC) analyzes and prioritizes the signal, assesses the signal, and, if needed, recommends regulatory action. The recommended actions are reported to people involved in medical services in order to give them more information about drugs and their safety. The new information generated by signal detection is used by MAHs to modify their risk-management plans (RMPs), which are documents required by EMA during the application for drug marketing. RMPs report the safety profile of a drug, explain how to prevent or minimize harm in patients taking the drug, and describe planned activities for learning more about the efficacy and safety of the drug and for measuring the effectiveness of those activities [6, 14].

PRAC also evaluates ADRs coming from post-authorization safety studies (PASS) and periodic safety update reports (PSUR). PASS are conducted after a drug enters the market, in order to identify, define, and quantify ADRs. Instead, PSUR are documents prepared by the MAHs to report an evaluation of the risk–benefit balance of a medicinal product [7, 15, 16]. All this information is used by EMA to produce recommendations, alerts, and measures to minimize the risk of ADRs.

The activities of pharmacovigilance are even more important for medicines under additional monitoring. *Additional monitoring* is required for drugs for which regulatory authorities require more information. In particular, it is applied to drugs that [17]: (1) contain an active substance authorized in the EU after 1 January 2011; (2) are biological medicines (vaccines and drugs derived from plasma); (3) have been given a conditional approval (the producer must provide more data about it) or have been approved under exceptional circumstances; (4) must be further studied so that the producer can provide long-term data on its safety; or (5) have been authorized with strict monitoring of ADRs. For drugs under additional monitoring, the SmPC document is marked by a black inverted triangle.

## EU pharmacovigilance terminology

According to a European directive from 2001 (2001/83/EC3) [18], a *medicinal product* (synonymous with “medicine” and “drug”) is:

(a) Any substance or combination of substances presented as having properties for treating or preventing disease in human beings; or (b) Any substance or combination of substances which may be used in or administered to human beings either with a view to restoring, correcting or modifying physiological functions by exerting a pharmacological, immunological or metabolic action, or to making a medical diagnosis.

In addition to their intended effects, medicinal products also often have unintentional effects, both positive and negative. These unintentional effects, when they occur at the normal pharmacological dosage, are called *side effects*. Generally, however, the term side effect is used to describe an adverse effect [19, 20]. An *adverse effect* (also called *adverse event*) is defined by EU Directive 2001/20/EC as “Any untoward medical occurrence in a patient or clinical trial subject administered a medicinal product and which does not necessarily have a causal relationship with this treatment” [21]. In other words, an adverse effect is not necessarily caused by the drug; it is only temporally related to the use of the product. An ADR is a form of adverse effect that is both temporally and causally related to the drug.

The EU’s definition of ADRs has changed over time. In Directive 2010/84/EU [22], an adverse reaction is defined as a “response to a medicinal product which is noxious and unintended”. The new definition also includes adverse reactions observed with non-authorized uses of the drug, specifically off-label use, medical errors, misuse, abuse, and occupational exposure. The EU’s definitions of these terms are given in Table 1.

**Table 1:** Terms used to describe adverse reactions observed with drugs used outside of authorized medicinal purposes, and their definitions according to the European Medicines Agency (EMA) [5]

Term	EMA definition
Abuse of a medicinal product	Persistent or sporadic, intentional excessive use of medicinal products which is accompanied by harmful physical or psychological effects [DIR 2001/83/EC Art 1(16)]
Medication error	An unintended failure in the drug treatment process that leads to, or has the potential to lead to, harm to the patient
Misuse of a medicinal product	Situations where a medicinal product is intentionally and inappropriately used not in accordance with the terms of the marketing authorisation
Occupational exposure to a medicinal product	For the purpose of reporting cases of suspected adverse reactions, an exposure to a medicinal product as a result of one’s professional or non-professional occupation It does not include the exposure to one of the ingredients during the manufacturing process before the release as finished product
Off-label use	Situations where a medicinal product is intentionally used for a medical purpose not in accordance with the terms of the marketing authorisation. Examples include the intentional use of a product in situations other than the ones described in the authorised product information, such as a different indication in terms of medical condition, a different group of patients (e.g. a different age group), a different route or method of administration or a different posology. The reference terms for off-label use are the terms of marketing authorisation in the country where the product is used
Overdose	Administration of a quantity of a medicinal product given per administration or cumulatively which is above the maximum recommended dose according to the authorised product information When applying this definition, clinical judgement should always be applied

Subtypes of ADRs have been defined. An *unexpected ADR* is an “adverse reaction, the nature, severity or outcome of which is not consistent with the summary of product characteristics (SmPC)” [9]. A *serious ADR* is an “adverse reaction which results in death, is life-threatening, requires in-patient hospitalisation or prolongation of existing hospitalisation, results in persistent or significant disability or incapacity, or is a congenital anomaly/birth defect” [21]. Finally, ADRs can be classified according to the type of reaction [23]:

- Type A: dose-related. These ADRs are related to the drug’s mechanism of action and are usually identified during clinical testing. They are common and predictable.
- Type B: non-dose related. These ADRs are related to the patient’s characteristics, and therefore are unexpected and uncommon. Generally, they are serious and with high mortality.
- Type C: dose- and time-related. These uncommon ADRs are caused by a cumulative effect derived from chronic use.
- Type D: time-related. These uncommon ADRs appear some time after the use of the drug.
- Type E: withdrawal. These ADRs appear soon after the end of the treatment and are uncommon.
- Type F: unexpected failure of therapy. These common, dose-related ADRs are caused by an unexpected failure of therapy, usually due to drug–drug interactions.

Every term to describe an adverse reaction (e.g. nausea, diarrhea) is defined in the clinically validated international *Medical Dictionary for Regulatory Activities* (MedDRA) [24]. This dictionary is used in every phase of drug development and after marketing authorization. In MedDRA, terminology is organized into five hierarchical levels called System Organ Class, High-Level Group Terms, High-Level Terms, Preferred Terms and Lowest Level Terms. The dictionary is used in signal detection to analyze particular adverse reactions (e.g. nausea), including those associated with an organ system (e.g. gastrointestinal). It also includes Standardized MedDRA Queries, which are groups of terms related to a defined medical condition or area of interest.

MedDRA is incorporated in the Common Terminology Criteria for Adverse Events (CTCAE) [25]. CTCAE is a terminology system for classifying and grading the severity of ADRs. In CTCAE, there are five grades (1–5). CTCAE helps understand the severity of an adverse event. In spontaneous reports, it is extremely important to report ADRs with their grade to allow the regulatory agencies to assess patients’ risk of harm when using the drug.

## Conclusions

There are many complex activities to ensure the safety of drugs. Clinical trials investigate ADRs with selected patients (age, sex, pathology, etc.), so pharmacovigilance is essential for observing ADRs in patients that take the drug as routine treatment and, consequently, for ensuring the safety of drugs. In order that ADRs are reported by health professionals and the pharmacovigilance process leads to useful consequences, it is important that the “actors” involved have a good knowledge of the terminology and processes of pharmacovigilance.
